# Retrograde Intrarenal Surgery versus Percutaneous Lithotripsy to Treat Renal Stones 2-3 cm in Diameter

**DOI:** 10.1155/2015/914231

**Published:** 2015-03-03

**Authors:** Kursad Zengin, Serhat Tanik, Nihat Karakoyunlu, Nevzat Can Sener, Sebahattin Albayrak, Can Tuygun, Hasan Bakirtas, M. Abdurrahim Imamoglu, Mesut Gurdal

**Affiliations:** ^1^Department of Urology, Medical Faculty, Bozok University, Yozgat, Turkey; ^2^Department of Urology, Ministry of Health, Diskapi Education and Research Hospital, Ankara, Turkey; ^3^Department of Urology, Ministry of Health, Numune Education and Research Hospital, Adana, Turkey

## Abstract

*Objective*. Retrograde intrarenal surgery (RIRS) performed using a flexible ureterorenoscope marked the beginning of a new era in urology. Today, even staghorn stones are successfully treated via RIRS. The recommended treatment for larger stones is percutaneous nephrolithotomy (PNL). However, the question of whether PNL or RIRS should be the first-line treatment option for larger stones remains controversial. In this study, we contribute to the debate by comparing the success and complication rates of PNL and RIRS that were used to treat renal pelvis stones 2-3 cm in diameter. *Materials and Methods*. The medical records of 154 patients (74 PNL, 80 RIRS) were retrospectively evaluated. PNL patients were placed in Group 1 and RIRS patients in Group 2. *Results*. The complete stone-free rates were 95.5% in the PNL group and 80.6% in the RIRS group 1 month postoperatively (*P* = 0.061). The respective complication rates (evaluated using the Clavien system) were 13.5% and 8.8% (*P* = 0.520). *Conclusions*. RIRS affords a comparable success rate, causes fewer complications than PNL, and seems to be a promising alternative to PNL when larger stones are to be treated. Prospective randomized controlled trials are needed to confirm these findings.

## 1. Introduction

Retrograde intrarenal surgery (RIRS) performed using a flexible ureterorenoscope marked the beginning of a new era in urology. RIRS renders smaller kidney stones more accessible and upper urinary tract tumors treatable, using minimally invasive methods [1]. RIRS was first used to treat small kidney stones [2]. The approach attracted a great deal of attention and it was suggested that larger stones could also be treated, albeit over longer operative times. Initially, medium and then larger stones were treated via RIRS [3].

The recommended treatment for larger stones is percutaneous nephrolithotomy (PNL) [4, 5], which affords very good success rates [6] but potentially causes high-level morbidity. Some urologists have suggested that RIRS, which is associated with fewer complications and less morbidity, should be used to treat large stones also. Indeed, the EAU guidelines mention that RIRS is the first choice of some surgeons who treat larger stones [4, 5].

Although PNL is an established method for treatment of renal stones, the complications are potentially hazardous. PNL may be associated with Grade 4 renal trauma [7]. In hemodynamically unstable patients with such trauma, either interventional radiology or open surgery is required. PNL can cause an arteriovenous fistula and/or a pseudoaneurysm, which must be treated with the aid of conventional radiology. Such potential complications intimidate urologists, especially those working in smaller institutions lacking interventional radiology departments. RIRS is safer when used to treat renal stones smaller than 2 cm in diameter. Clinically, we notice that when we explain the potential complications of PNL to patients, to obtain informed consent, most patients ask if a safer procedure is available. Some patients preferred RIRS even though they were told that more than one operative session might be required, especially if the stones were large. Also, the legal aspects of surgical procedures require constant attention. All surgeons and patients prefer minimally invasive surgical solutions, which are safer and associated with lower complication rates.

We compared RIRS and PNL that were used to treat larger kidney stones. Specifically, we compared the success rates and complications of these two minimally invasive methods that were used to treat kidney stones 2-3 cm in diameter.

## 2. Materials and Methods

### 2.1. Study Population

Between September 2012 and August 2014, 164 patients with renal pelvic stones 2-3 cm in diameter were treated in our department. Patients with histories of ipsilateral kidney operations, ureteropelvic junction obstructions, and/or failed shock wave lithotripsy (SWL) and/or who were undergoing concomitant surgery (e.g., endopyelolithotomy) were excluded.

The medical records of 154 patients (74 PNL, 80 RIRS) were retrospectively evaluated. Patients treated using PNL constituted Group 1 and those treated via RIRS Group 2. All patients in each group were treated by a single surgeon (thus, two surgeons treated all patients).

### 2.2. Operative Techniques

#### 2.2.1. F-URS Technique

All F-URS procedures were performed under general anesthesia with patients in the lithotomy position. Prior to flexible ureteroscopy, rigid ureteroscopy was routinely performed to passively dilate the ureter and to place a hydrophilic safety guidewire (0.038-inch) that was advanced to the renal pelvis with fluoroscopic assistance. Next, a ureteral access sheath (11/13 F) was passed over the guidewire through the ureteropelvic junction. A flexible ureterorenoscope (Flex-X2, Karl Storz, Tuttlingen, Germany) was inserted into the renal pelvis within the ureteral access sheath. Kidney stones were fragmented to dust with the aid of a holmium laser (Ho YAG Laser; Dornier MedTech, Munich, Germany).

#### 2.2.2. PNL Technique

**Figure 1:**
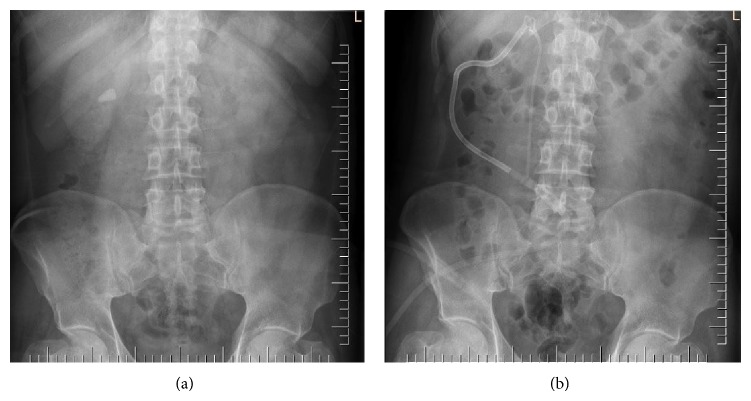
Preoperative (a) and postoperative (b) X-rays of a patient with a 21 mm diameter kidney stone treated via percutaneous nephrolithotomy.

A ureteral catheter was placed, via rigid cystoscopy, with the patient in the lithotomy position. Next, percutaneous access was achieved with the aid of a C-arm fluoroscopic device, with the patient in the prone position, using an 18-gauge needle and a guidewire. The ureter was dilated up to 30 F using Amplatz dilators. Stones were fragmented using a pneumatic lithotripter (LithoClast; EMS, Nyon, Switzerland) and retrieval graspers inserted through a rigid nephroscope (26 F, Karl Storz). A nephrostomy tube was placed at the end of the procedure (Figure 1). Tubes were removed on postoperative days 1-2 and patients were discharged home the next day.

### 2.3. Outcomes

The groups were compared in terms of stone diameters, success rates, operative times, intraoperative fluoroscopy times, mean decreases in hemoglobin levels, differences between preoperative and postoperative serum creatinine levels, and complication rates, using the modified Clavien grading system. Also, hospital stays (in days) were compared.

All patients underwent low-dose helical computed tomography (CT) of the abdomen prior to operation. Patients were reevaluated using CT 1 month after surgery to determine residual stone status. Residual stones <2 mm in diameter were considered to be “clinically insignificant residues.”

## 3. Results

Mean patient age was 45.6 years in Group 1 and 48.2 years in Group 2 (*P* = 0.546). Mean stone diameters were similar in both groups (2.6 ± 0.3 cm versus 2.3 ± 0.4 cm, *P* = 0.151).

In Group 1, 71 patients were stone-free 1 month postoperatively; the figure for Group 2 was 65. The complete stone-free rate was 95.5% in the PNL group and 80.6% in the RIRS group 1 month postoperatively (*P* = 0.061). Two patients of Group 1 had clinically insignificant residual stones, as did six patients of Group 2. The clinically insignificant residual stone (<2 mm) rate was thus 2.7% in Group 1 and 7.5% in Group 2 (*P* = 0.471). The residual stone (≥2 mm) rate was 11.2% in the RIRS group, but no significant residual stones were noted in the PNL group.

The mean operative time was 63 ± 22 min in the PNL group and 81 ± 41 min in the RIRS group (*P* < 0.001). The mean fluoroscopy time was 38 ± 14 s in the PNL group and 18 ± 9 s in the RIRS group (*P* < 0.001). The mean decrease in hemoglobin level was 1.4 ± 0.9 g/dL in the PNL group and 0.3 ± 0.1 g/dL in the RIRS group (*P* < 0.001). The mean difference between the postoperative and preoperative creatinine levels was 0.24 ± 0.19 mg/dL in the PNL group and 0.11 ± 0.08 mg/dL in the RIRS group (*P* = 0.039). The mean hospital stay was 2.3 ± 1.3 days in the PNL group and 1.1 ± 0.4 days in the RIRS group (*P* = 0.032). These data are summarized in Table 1. Complication rates determined using the Clavien grading system were 13.5% in the PNL group and 8.8% in the RIRS group (*P* = 0.520) (Table 2).

## 4. Discussion

The European Association of Urology urolithiasis guidelines recommend PNL as first-line therapy for treatment of large kidney stones [4, 5].

Haggag et al. used PNL to treat 40 patients with renal pelvis stones 2.5 cm or greater in diameter. The stone-free rate was 80% [8]. Our stone-free rate was 95.5%, thus higher than that of Haggag et al., attributable to the fact that the stones of our cohort were larger than those of the patients treated by Haggag et al.

Singh et al. treated renal pelvis stones >3 cm in diameter via either PNL or retroperitoneoscopic pyelolithotomy. Each group contained 22 patients. The stone-free rate was 72.7% 1 day postoperatively and 95% 3 months later [9]. Zeng et al. used minimally invasive PNL (featuring small tracts and instruments) to perform 13,984 procedures over 20 years. The mean stone diameter was 3.2 ± 0.8 cm and the stone-free rate was 78.6%. However, after “second looks,” that rate increased to about 90% [10]. Thus, the success rate was lower than ours. The principal difference between the two studies is that Zeng et al. created smaller tracts to reduce morbidity. We used RIRS instead of PNL, to the same end, and our success rate was good. We believe that RIRS is a suitable alternative when low morbidity is prioritized.

Giusti et al. treated kidney stones >2 cm in diameter via RIRS. A total of 162 patients had an average stone diameter 2.7 ± 0.6 cm. The success rate was 87.7% with an average of 1.48 operative sessions per patient. RIRS was considered to be safe and effective when used to treat kidney stones >2 cm in diameter [11]. Hyams et al. used RIRS to treat 120 patients with kidney stones 2-3 cm in diameter. Of these, 63% had residual stones <2 mm in diameter and 83% residual stones <4 mm in diameter. The complication rate was 6.7%, and 78% of patients were treated in the outpatient clinic [12]. Bryniarski et al. compared PNL and RIRS that were used to treat kidney stones >2 cm in diameter. Each group had 32 patients; the success rates were 94% in the PNL group and 75% in the RIRS group [13]. Akman et al. compared RIRS and PNL that were used to treat kidney stones 2–4 cm in diameter. Similar to what was found by Bryniarski et al., the success rate in the RIRS group was 73.5% compared to 91.2% in the PNL group [14]. The RIRS success rate was similar to ours (80.6%). The cited authors also recommended RIRS as an alternative to PNL, when kidney stones >2 cm in diameter were to be treated.

Fluoroscopy time is important when choosing the optimal treatment. Prolonged exposure to X-rays harms both surgeon and patient. The protective maxim used is termed ALARA ([exposure is to be] as low as reasonably achievable) [15]. PNL is associated with greater exposure to X-rays than is RIRS. Reduced X-ray exposure renders patients less prone to falls in hemoglobin levels and is associated with shorter hospital stays. Thus, RIRS has certain advantages compared to PNL. However, PNL is associated with considerably higher stone-free rates and shorter operative times.

Several investigators have attempted to maximize the efficacy of methods used to treat large-diameter kidney stones and enhance safety. Miernik et al. [16] combined flexible ureterorenoscopy with placement of a ureteral access sheath with a large-diameter lumen and semirigid ureteroscopy, to treat kidney stones >2 cm in diameter. The stone-free rate was comparable to that attained via PNL, and the cited authors concluded that their combination therapy could serve as an alternative first-line therapy. Hamamoto et al. combined RIRS with mini-PNL, to exploit the advantages of either method. Of the three groups of patients, one arm underwent mini-PNL, one arm RIRS, and one the combination therapy. The latter group experienced shorter operative times and the stone-free rate was the highest of all three groups [17].

Several limitations of our study are apparent. These are the retrospective nature of our work, the relatively small patient cohort, and the lack of randomization. However, we believe that we have addressed an important “grey area” of daily urological practice.

## 5. Conclusion

In patients with renal pelvis stones 2-3 cm in diameter, PNL has been regarded as the optimum method. However, RIRS affords a comparable success rate, causes fewer risks of complications, and seems to be a promising alternative to PNL when larger stones are to be treated. Prospective randomized controlled trials are needed to confirm these findings.

## Figures and Tables

**Table 1:** Patient demographics and operative parameters.

	Group 1	Group 2	*P*
Age (years)	45.6	48.3	0.546
Stone diameter (cm)	2.6	2.3	0.151
Gender (M/F)	34/40	38/42	
BMI (kg/m^2^)	30.35	31.58	0.095
Stone-free rate (%)	95.5	80.6	0.061
Operative time (min)	63	81	<0.001
Fluoroscopy time (s)	38	18	<0.001
Change in creatinine level (mg/dL)	0.24	0.11	0.039
Decrease in hemoglobin level (mg/dL)	1.4	0.3	<0.001
Hospital stay (days)	2.3	1.1	0.032

**Table 2:** Complications assessed using the modified Clavien grading system.

	Group 1	Group 2
Grade 1	5	3
Grade 2	3	4
Grade 3		
A	1	0
B	1	0
Grade 4		
A	0	0
B	0	0
Grade 5	0	0

Total	10 (8.8%)	7 (13.5%)

## References

[1] Abdel-Razzak O. M., Bagley D. H. (1992). Clinical experience with flexible ureteropyeloscopy. *The Journal of Urology*.

[2] Bagley D. H. (2002). Expanding role of ureteroscopy and laser lithotripsy for treatment of proximal ureteral and intrarenal calculi. *Current Opinion in Urology*.

[3] Breda A., Ogunyemi O., Leppert J. T., Lam J. S., Schulam P. G. (2008). Flexible ureteroscopy and laser lithotripsy for single intrarenal stones 2 cm or greater—is this the new frontier?. *Journal of Urology*.

[4] Turk C., Knoll T., Petrik A., Sarika K., Straub M. (2001). Guidelines on urolithiasis. *European Urology*.

[5] Arbeitskreis Harnsteine der Akademie der Deutschen Urologen1, Arbeitskreis Endourologie und Steinerkrankung der Osterreichischen Gesellschaft für Urologie, Knoll T. (2009). S2 guidelines on diagnostic, therapy and metaphylaxis of urolithiasis: part 1: diagnostic and therapy. *Der Urologe. Ausg. A*.

[6] Gök A., Gunes Z., Kilic S., Gök B., Yazicioglu A. (2014). Factors influencing the duration of fluoroscopy in percutaneous nephrolithotomy. *Journal of Clinical and Analytical Medicine*.

[7] Egilmez T., Goren M. (2015). Predicting surgical outcome of percutaneous nephrolithotomy: validation of the Guy's stone score and nephrolithometric nomogram in terms of success and complications. *Journal of Clinical and Analytical Medicine*.

[8] Haggag Y. M., Morsy G., Badr M. M., Al Emam A. B. A., Farid M., Etafy M. (2013). Comparative study of laparoscopic pyelolithotomy versus percutaneous nephrolithotomy in the management of large renal pelvic stones. *Journal of the Canadian Urological Association*.

[9] Singh V., Sinha R. J., Gupta D. K., Pandey M. (2014). Prospective randomized comparison of retroperitoneoscopic pyelolithotomy versus percutaneous nephrolithotomy for solitary large pelvic kidney stones. *Urologia Internationalis*.

[10] Zeng G., Mai Z., Zhao Z. (2013). Treatment of upper urinary calculi with Chinese minimally invasive percutaneous nephrolithotomy: a single-center experience with 12,482 consecutive patients over 20 years. *Urolithiasis*.

[11] Giusti G., Proietti S., Luciani L. G. (2014). Is retrograde intrarenal surgery for the treatment of renal stones with diameters exceeding 2 cm still a hazard?. *Canadian Journal of Urology*.

[12] Hyams E. S., Munver R., Bird V. G., Uberoi J., Shah O. (2010). Flexible ureterorenoscopy and holmium laser lithotripsy for the management of renal stone burdens that measure 2 to 3 cm: a multi-institutional experience. *Journal of Endourology*.

[13] Bryniarski P., Paradysz A., Zyczkowski M., Kupilas A., Nowakowski K., Bogacki R. (2012). A randomized controlled study to analyze the safety and efficacy of percutaneous nephrolithotripsy and retrograde intrarenal surgery in the management of renal stones more than 2 cm in diameter. *Journal of Endourology*.

[14] Akman T., Binbay M., Ozgor F. (2012). Comparison of percutaneous nephrolithotomy and retrograde flexible nephrolithotripsy for the management of 2-4 cm stones: a matched-pair analysis. *BJU International*.

[15] Söylemez H., Altunoluk B., Bozkurt Y., Sancaktutar A. A., Penbegül N., Atar M. (2012). Radiation exposure-do urologists take it seriously in Turkey?. *Journal of Urology*.

[16] Miernik A., Schoenthaler M., Wilhelm K. (2014). Combined semirigid and flexible ureterorenoscopy via a large ureteral access sheath for kidney stones >2 cm: a bicentric prospective assessment. *World Journal of Urology*.

[17] Hamamoto S., Yasui T., Okada A. (2014). Endoscopic combined intrarenal surgery for large calculi: simultaneous use of flexible ureteroscopy and mini-percutaneous nephrolithotomy overcomes the disadvantageous of percutaneous nephrolithotomy monotherapy. *Journal of Endourology*.

